# Four new Mouse Spider species (Araneae, Mygalomorphae, Actinopodidae, *Missulena*) from Western Australia

**DOI:** 10.3897/zookeys.410.7156

**Published:** 2014-05-22

**Authors:** Laura Tavares Miglio, Danilo Harms, Volker Wilhelm Framenau, Mark Stephen Harvey

**Affiliations:** 1 Laboratório de Aracnologia, Museu Paraense Emílio Goeldi. Caixa Postal 399, 66017−970 Belém, PA, Brazil; 2 School of Animal Biology, The University of Western Australia, 35 Stirling Highway, Crawley, Western Australia 6009, Australia; 3 Department of Terrestrial Zoology, Western Australian Museum, Locked Bag 49, Welshpool DC, Western Australia 6986, Australia; 4 Phoenix Environmental Sciences Pty Ltd, 1/511 Wanneroo Road, Balcatta, Western Australia 6021, Australia; 5 School of Natural Sciences, Edith Cowan University, Joondalup, Western Australia 6027, Australia; 6 Division of Invertebrate Zoology, American Museum of Natural History, 79th Street @ Central Park West, New York, New York 10024–5192, USA; 7 Department of Entomology, California Academy of Sciences, Golden Gate Park, San Francisco, California 94103–3009 USA

**Keywords:** Biodiversity, Gondwanan fauna, systematics, taxonomy, trapdoor spiders

## Abstract

Four new species of the Mouse Spider genus *Missulena* Walckenaer, 1805 (family Actinopodidae) are described from Western Australia based on morphological features of adult males. *Missulena leniae* **sp. n.**(from the Carnarvon and Yalgoo biogeographic regions), *Missulena mainae* **sp. n.** (Carnarvon), *Missulena melissae* **sp. n.** (Pilbara) and *Missulena pinguipes* **sp. n.** (Mallee) represent a broad spectrum of morphological diversity found in this genus and differ from other congeners by details of the male copulatory bulb, colour patterns, eye sizes, leg morphology and leg spination. Two of the species, *M. pinguipes* **sp. n.** and *M. mainae* **sp. n.**, are characterised by swollen metatarsi of the fourth legs in males, a feature not previously recorded in the family. A key to males of all named *Missulena* species from Australia is presented and allows their identification based on external morphology.

## Introduction

The mygalomorph spider family Actinopodidae Simon, 1892 has a southern Gondwanan distribution with species found in the Neotropical and Australasian regions, specifically the tropical and temperate zones of Australia, South and Central America. The family includes 42 described species in three genera: the Neotropical *Actinopus* Perty, 1833 (27 described species), *Missulena* Walckenaer, 1805 from Australia and Chile (13 species), and the exclusively Chilean genus *Plesiolena* Goloboff & Platnick, 1987 (2 species) (Platnick 2014).

Species belonging to *Missulena*, commonly referred to as Mouse Spiders, are amongst the most recognisable Australian arachnids, with males of *Missulena insignis* (O. Pickard-Cambridge, 1877), *Missulena langlandsi* Harms & Framenau, 2013, *Missulena occatoria* Walckenaer, 1805 and *Missulena reflexa* Rainbow & Pulleine, 1918 displaying conspicuous red fangs and cephalic areas. Adult males wander during the day, adding to their prominent status amongst naturalists and resulting in their frequent illustration in field guides (e.g. Brunet 1994; Brunet 2000; Mascord 1970). Not all species are colourful and the red markings can be reduced, e.g. in *Missulena hoggi* Womersley, 1943, or entirely absent as in *Missulena dipsaca* Faulder, 1955, *Missulena granulosa* (O. Pickard-Cambridge, 1869), *Missulena faulderi* Harms & Framenau, 2013, *Missulena rutraspina* Faulder, 1995 and *Missulena torbayensis* Main, 1996. Mouse spiders have also received attention due to the toxicity of their venom that appears to be similar in composition to that of Australian funnel-web spiders (family Hexathelidae); however, severe cases of envenomation are rare and not all species appear equally harmful (Herzig et al. 2008; Isbister 2004; Rash et al. 2000).

Several species, namely the type species *Missulena occatoria*, but also *Missulena granulosa* and *Missulena insignis*, were amongst the first spiders to be collected and described from Australia, resulting in a confusing taxonomic history because early type localities were not recorded (e.g. “New Holland” for the nominate species *Missulena occatoria*), old taxonomic descriptions were poor by modern standards, and some type specimens appear to be lost (Main 1985).

The first attempt towards a more integrated taxonomy (Womersley 1943) recognised six species, only four of which are known from both male and female specimens. Little taxonomic work was undertaken subsequently, with the description of *Missulena pruinosa* from the Northern Territory by Levitt-Gregg (1966). Main (1985) catalogued all species, summarised the taxonomic literature and provided preliminary distribution data. Her contribution stimulated several subsequent taxonomic papers, all adding additional species from Western Australia: *Missulena dipsaca*, *Missulena rutraspina* (both Faulder 1995b), *Missulena torbayensis* (Main 1996), and more recently *Missulena faulderi* and *Missulena langlandsi* (both Harms and Framenau 2013). It was also after the publication of Main’s (1985) catalogue that that the first Chilean representative, *Missulena tussulena* Goloboff, 1994, was described.

In addition to taxonomic advances, the major phylogenetic treatises of Raven (1985) and Goloboff and Platnick (1987) developed a diagnosis of *Missulena* against other mygalomorph spiders: the anterior row of eyes is almost straight, the posterior median eyes are closer to the anterior lateral eyes than the posterior lateral eyes, the male pedipalps are shorter than the first leg, the patella of the first leg has robust spines, the sternum is rebordered, and the male pedipalp embolus is almost straight and thinner than that of other Actinopodidae.

*Missulena* currently has the highest species diversity in Western Australia where ten of the 12 Australian species occur, six of which are endemic to the state (Table 1). Recent large-scale environmental surveys conducted in Western Australia (e.g. Durrant et al. 2010; Main et al. 2000) have discovered additional morphospecies and confirm the previous notion that the diversity of this genus is underrepresented by the current taxonomy (Harms and Framenau 2013; Main 1985). It is clear, that the species of Australian *Missulena* known to date represent merely a fraction of the actual species diversity in this region.

**Table 1.** Distribution of *Missulena* species in Australia.

Species	Distribution	Remarks/selected source^1^
*Missulena bradleyi* Rainbow, 1914	Qld, NSW, Vic	Faulder (1995b), Raven and Seeman (2008), Walker et al. (2003)
*Missulena dipsaca* Faulder, 1995	NSW, Vic, SA, WA, Qld	Faulder (1995b)
*Missulena faulderi* Harms & Framenau, 2013	WA	Harms and Framenau (2013)
*Missulena granulosa* (O. Pickard-Cambridge, 1869)	WA	Faulder (1995a)
*Missulena hoggi* Womersley, 1943	WA	Womersley (1943)
*Missulena insignis* (O. Pickard-Cambridge, 1877)	WA	Faulder (1995a)
*Missulena langlandsi* Harms & Framenau, 2013	WA	Harms and Framenau (2013)
*Missulena leniae* sp. n.	WA	This study
*Missulena mainae* sp. n.	WA	This study
*Missulena melissae* sp. n.	WA	This study
*Missulena occatoria* Walckenaer, 1805	NSW, Qld, Vic, ACT, SA, NT, WA	Type locality unknown; Faulder (1995a)
*Missulena pinguipes* sp. n.	WA	This study
*Missulena pruinosa* Levitt-Gregg, 1966	Qld, WA, NT	Raven and Seeman (2008), Faulder (1995a)
*Missulena reflexa* Rainbow & Pulleine, 1918	SA	Womersley (1943)
*Missulena rutraspina* Faulder, 1995	WA, SA, Vic	Faulder (1995b)
*Missulena torbayensis* Main, 1996	WA	Main (1996)

^1^ published literature and university theses only

The aim of this paper is twofold. Firstly, we add four new species of *Missulena* to the currently described Australian fauna, resulting in a total of 16 species (Table 1). These new species have very distinctive male morphologies and differ clearly from all other named species although the taxonomic status of some of these remains poorly resolved. Secondly, we provide a key that aids in the identification of males of all described species. A comprehensive key has not been published since Womersley (1943), although most species were described since then.

A comprehensive revision of *Missulena*, which includes a considerable undocumented fauna is beyond the scope of this study as it would require substantial funding and full-time commitment.

## Material and methods

### Morphology

Specimens used for morphological examination were preserved in 75% ethanol. Material was examined using a Leica MZ16A stereomicroscope. Digital images were taken using a Leica DFC 500 digital camera attached to a Leica MZ16A stereomicroscope controlled by the Leica Application Suite Version 3.7. This program allows the alignment of images taken at different focal planes (here ca. 20–40 images) and combines them into a single image. The images were edited and formatted in Adobe Photoshop, version CS5.

The specimens examined for this study are lodged in the Western Australian Museum, Perth, Australia (WAM). We also examined type material of all Australian *Missulena* that was available to us (Table 2).

**Table 2.** Type material of Australian *Missulena* examined for this study.

Species	Type	Location and repository
*Missulena bradleyi* Rainbow, 1914	holotype male allotype female	North Sydney (NSW) (AM KS6402), Willoughby (NSW) (AM KS6401)
*Missulena dipsaca* Faulder, 1995	holotype male	Junee (NSW) (AM KS9308)
*Missulena faulderi* Harms & Framenau, 2013	holotype male paratype male	Jinayri (WA) (WAM T97017), Jinayri (WA) (WAM T96132)
*Missulena langlandsi* Harms & Framenau, 2013	holotype male paratype male	Newman (WA) (WAM T115948), Newman (WA) (WAM T112076)
*Missulena pruinosa* Levitt-Gregg, 1966	holotype male	Groote Eylandt Island (NT) (AM KS6403)
*Missulena reflexa* Rainbow & Pulleine, 1918	holotype male	Keith (SA) (AM KS6404)
*Missulena torbayensis* Main, 1996	holotype male	Torbay (WA) (WAM 95/2)

The distribution data for species is described within the context of the Interim Biogeographic Regionalisation for Australia (IBRA) (Department of the Environment 2013).

All measurements are expressed in millimetres. The format of the descriptions and measurements follows Griswold and Ledford (2001), except for the spination pattern of the legs that is described according to Harms and Framenau (2013). Spine counts were taken from the right legs. The number of teeth on the claws is given as the formula “leg number: number of teeth of lateral claws/number of teeth of median claw”. The leg formula is given as the order of the leg lengths from longest to shortest. The leg “index” is given here as the leg length divided by carapace length and indicates the ratio of leg lengths versus carapace. The term “rasps” refers to the presence of short but strong conical spines on the patellae of all legs. The presence of such rasps on patella I is a potential synapomorphy for *Missulena* species (Goloboff and Platnick 1987).

The following abbreviations were used:

*Morphology*: (EL) embolar lamella, (DET) distal embolar tooth, (BEI) basal embolar intumescence, (d) dorsal, (v) ventral, (p) prolateral, (r) retrolateral, (PME) posterior median eyes, (PLE) posterior lateral eyes, (ALE) anterior lateral eyes, (AME) anterior median eyes, (MOQ) median ocular quadrangle, (OAL) ocular area length, (OAW) ocular area width, and (HF) height from the fovea.

*Distribution*: (NSW) New South Wales, (Qld) Queensland, (Vic) Victoria, (SA) South Australia, (WA) Western Australia, (ACT) Australian Capital Territory, (NT) Northern Territory.

*Museums*: (WAM) Western Australian Museum, (AM) Australian Museum.

The taxonomic key is based on a complete inventory of the available literature and examination of type material of many species. We have restricted this key to males because nine of the now 16 Australian described species are known from the male gender only; females remain unknown and are morphologically less distinct. We note that this key is preliminary because many additional unnamed species are present in collections, at least from Western Australia.

## Systematics

### Family Actinopodidae Simon, 1892

#### *Missulena* Walckenaer, 1805

*Missulena* Walckenaer, 1805: 8. Type species: *Missulena occatoria* Walckenaer, 1805, by monotypy.

*Eriodon* Latreille, 1806: 85. Type species: *Eriodon occatorius* Latreille, 1806, by monotypy.

##### Missulena melissae sp. n.

http://zoobank.org/ABC49948-F3B9-4F6C-9C86-67B18B20605A

http://species-id.net/wiki/Missulena_melissae

**Figure 1.**
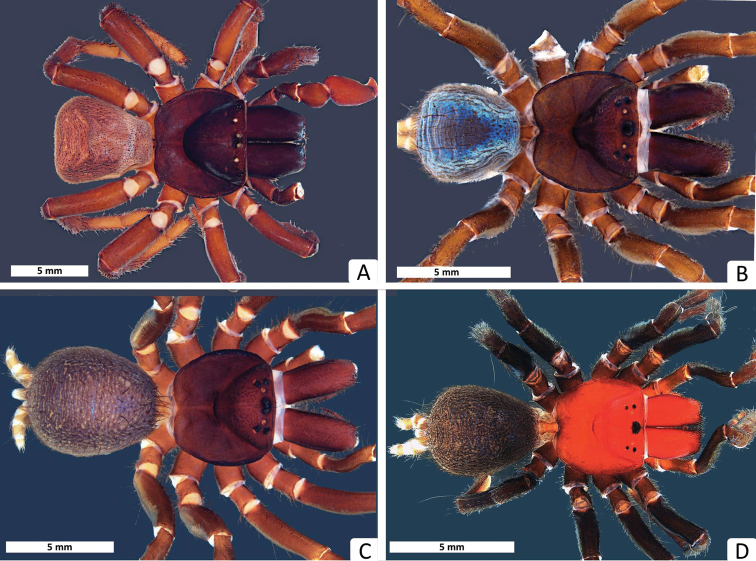
Dorsal habitus of species described in this paper: **A** *Missulena melissae* sp. n., holotype male from Millstream-Chichester National Park (WAM T97323) **B** *Missulena pinguipes* sp. n., holotype male from Digger Rocks (WAM T92331) **C** *Missulena leniae* sp. n., holotype male from Nanga Station, Shark Bay (WAM T96784) **D** *Missulena mainae* sp. n., holotype male from Quobba Station, Cape Cuvier (WAM T96782).

**Figure 2.**
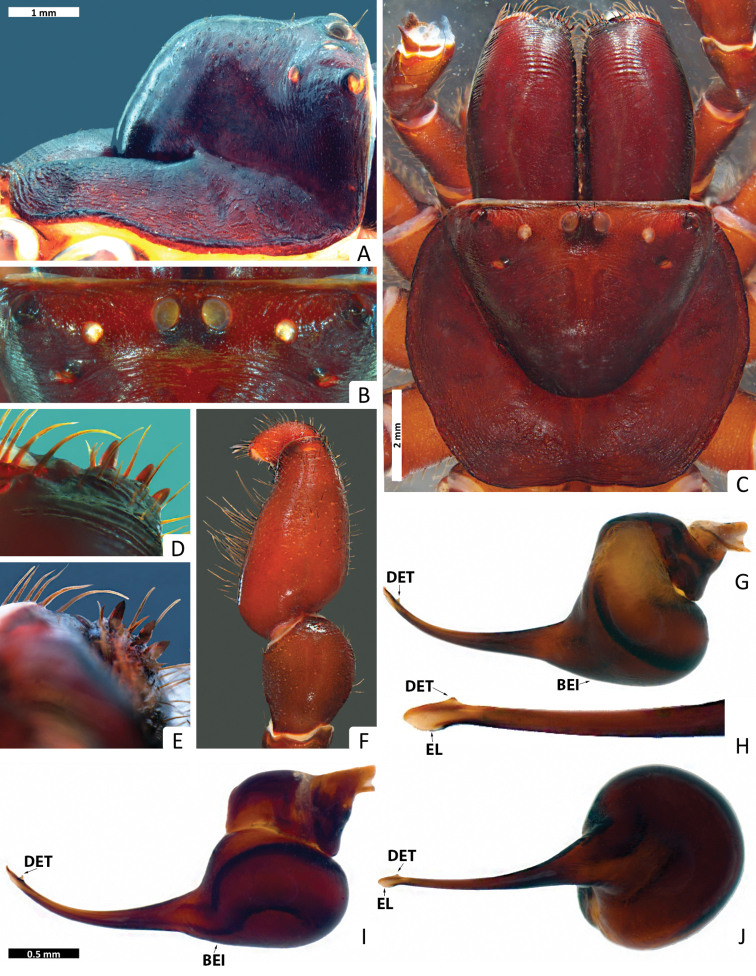
*Missulena melissae* sp. n., holotype male (WAM T97323): **A** carapace, lateral view **B** eye group, dorsal view **C** carapace, dorsal view **D** rastellum, dorsal view **E** same, ventral view **F** pedipalp, proventral view **G** bulb and embolus, retrolateral view **H** embolus, ventral view **I** bulb and embolus, prolateral view **J** same, ventral view. Arrows: (EL) embolar lamella, (DET) distal embolar tooth, and (BEI) basal embolar intumescence.

**Figure 3.**
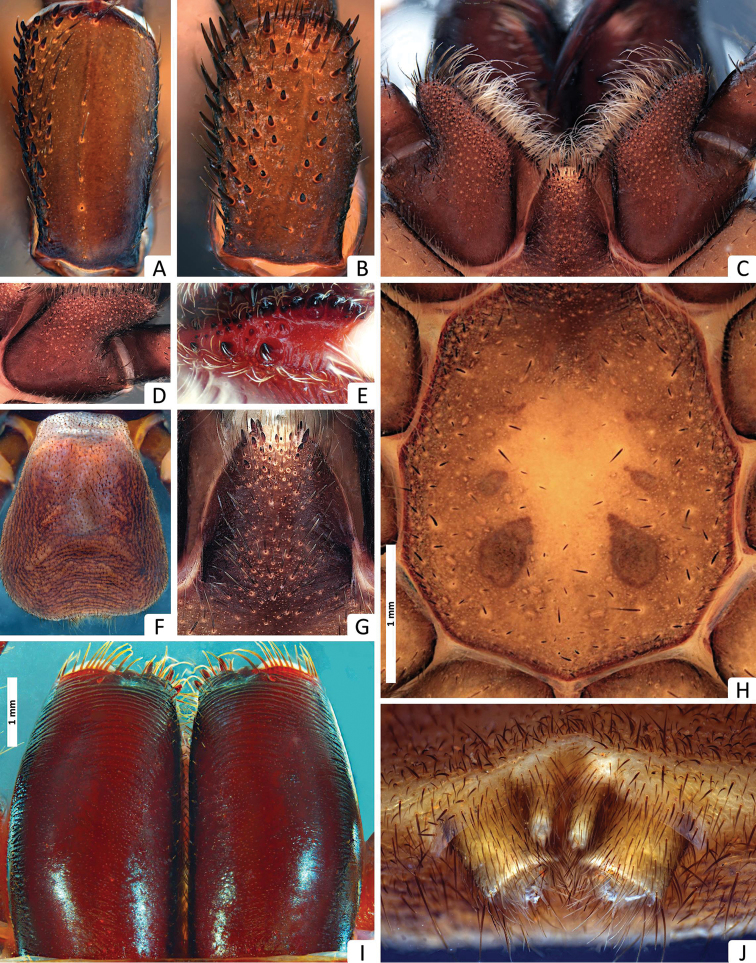
*Missulena melissae* sp. n., holotype male (WAM T97323): **A** patella II, dorsal view **B** patella III, dorsal view **C** coxae and labium of pedipalp, ventral view **D** coxae of pedipalp, ventral view **E** cheliceral groove, retroventral view **F** abdomen, dorsal view **G** labium, ventral view **H** sternum and sigillae, ventral view **I** chelicerae, dorsal view **J** spinnerets, ventral view.

Figs 1A , 2A–J , 3A–J

###### Type material.

**AUSTRALIA:** ***Western Australia*:** holotype male, Millstream-Chichester National Park, 6 km N. of Millstream Homestead, site PW11, 21°32'24.8"S, 117°03'25.2"E, 15 July 2003−11 October 2004, wet pitfall trap, Department of CALM staff (WAM T97323). Paratypes: 2 males, same data as holotype, except Corunna Downs, 52.5 km N. of Nullagine, Pilbara Biological Survey site NW11, 21°24'27.7"S, 120°04'16.7"E, 3 August 2003−20 October 2004, Department of CALM staff (WAM T120931).

###### Etymology.

The specific epithet is a patronym in honour of Melissa Thomas, the third author’s partner, for her continuing support of late-night arachnological endeavours.

###### Diagnosis.

Males of *Missulena melissae* sp. n. differ from the two other species with a brown body colour, strongly-sclerotised rastellum with thick spines, smooth and glabrous chelicerae with prominent horizontal ridges and short claws (i.e. *Missulena faulderi* and *Missulena rutraspina*) by details of the bulb and somatic morphology: embolus short and with a distal tooth (exceeding length of the bulb and without distal tooth in *Missulena faulderi*), carapace length ca. 4 cm (3.5 cm in *Missulena rutraspina*), patella I-III with rasps (patella III only in *Missulena faulderi*), rastellum on a low mound (mound distinct in *Missulena faulderi*), inner row of cheliceral teeth divided (first six teeth fused in *Missulena faulderi*), and pedipalp patella and tibia swollen (much more slender in *Missulena faulderi*). Males of *Missulena rutraspina* differ in having a simple embolus tip without processes, a straight embolus, rasps on patella III only, rastellum on a distinct mound, inner row of cheliceral teeth with six spaced teeth, and pedipalp tibia and patella more slender. The female of *Missulena melissae* sp. n. is unknown.

###### Description.

Adult male, based on holotype (WAM T97323). Medium-sized mygalomorph spider (total length 8.00).

*Colour*: carapace (Fig. 1A) dark reddish-brown, margins dark brown; eye region (Fig. 2B) dark brown, anterior median eyes on black tubercle; chelicerae (Fig. 3I) dark reddish-brown, fangs reddish-brown; abdomen (Fig. 1A) pale brown with a grey metallic spot in dorsal region; sternum (Fig. 3H) yellowish-brown, margins contoured dark yellowish-brown, sigillae yellowish-brown; labium (Fig. 3G) and maxillae (Fig. 3C) dark reddish-brown; legs (Figs 1A, 3A–B) yellowish brown, tarsi and metatarsi ventrally yellow; spinnerets (Fig. 3J) pale gray, spigots white.

*Carapace*: 3.81 long, 4.0 wide; clypeus 0.13; fovea 2.18; caput and eye region (Fig. 2A–C) laterally elevated, strongly arched; fovea (Fig. 2A, C) very deep, wide and strongly procurved, medially extending as triangular depression, pars cephalica smooth around the eyes and between the eyes and fovea, pars thoracica rugose with bands of fine, random fissures centered around fovea (Fig. 2C).

*Eyes*: OQ 3.61 times wider than long, occupying 1.12 of cephalic width; OAW 3.54; OAL 2.68; IPF 0.40; width of anterior eye group 2.95, with of posterior group 2.36, OQ length 0.81; PME 0.19; PLE 0.22; ALE 0.26; AME 0.22, AME on tubercle, 0.27 long, 0.62 wide; AME inter-distance 0.65; AME to ALE 1; AME to PME 0.40; PLE to ALE 0.55; PLE to PME 0.41; PME inter-distance 1.41; PME to ALE 0.55; eye region (Fig. 2B) with reduced setation although some setae present anterior to AME and between posterior eyes and fovea.

*Chelicerae*: 2.95 long, 1.54 wide; distally broad, diagonal, slightly conical; edges smoothly rounded; with weak transverse ridges which distally extend over entire length (Fig. 3I), without setae in area of transverse ridges but with ca. 60 short setae along inner margin of chelicera; rastellum developed, slightly pronounced, consisting of a sclerotised process with 3 strong conical spines and 12−14 disordered setae (Fig. 3I), 12−13 long setae extend forward from anterior margin of each chelicera and cover base of fang, setae largest on latero-ventral side; inner margin of cheliceral furrow with 3 rows of teeth (Fig. 3E); prolateral (inner) row with ca. 12 teeth, 3 proximal teeth fused together and the rest spaced; intermediate row with 9 proximal, spaced teeth; retrolateral (outer) row with 3 proximal, spaced teeth.

*Maxillae*: 1.86 long; 1.31 wide, longer than wide (Fig. 3C, D), with ca. 57 pointed cuspules along entire anterior margin, distally pointed and extended into a prominent heel.

*Labium*: 1.04 long, 0.77 wide; conical, ca. 20 pointed cuspules anteriorly (Fig. 3G); labiosternal suture developed as a shallow groove; a pair of sigilla near labiosternal suture (Fig. 3H), developed as irregular, poorly-defined patches.

*Sternum*: 2.27 long, 2.09 wide; oval and rebordered (Fig. 3H), with prominent setae, arranged irregularly but denser lateral to labium; 4 pairs of sigillae, anterior and second pair (anterior-posterior) smallest and poorly defined, third pair bigger than 2 anterior pairs and poorly defined, and posterior pair bigger than all others, roughly oval and well defined, 3 posterior sigillae slightly depressed.

*Abdomen*: 4 long, 3.54 wide; roughly oval (Fig. 3F); 4 spinnerets (Fig. 3J), PLS 0.72 long, 0.45 wide, apical segment domed; PMS 0.40 long, 0.13 wide.

*Pedipalp*: length of trochanter 0.85, femur 2.11, patella 0.33, tibia 0.66, tarsus 0.18; entire palp is aspinose, femur more than 3 times longer than tibia, tarsus terminally blunt (Fig. 2F); bulb pyriform and rather stout than globular (Fig. 2G, I−J), 2 strongly sclerotised sections connected by a velar median structure (“haematodocha”, Fig. 2G, I); bulb strongly twisted proventrally (Fig. 2I); embolus short, with an intumescence in proximal region (BEI), a strong curvature in the duct in prolateral view, tapering and slightly twisted medially (Fig. 2I, J); embolus tip triangular, with a lamella (EL) well-developed and a prominent tooth (DET) in all views (Fig. 2G–J).

*Legs*: with few brown setae, ventral setae of tibiae and metatarsi generally much longer and thicker than dorsal setae and bent towards the exterior; dorsal, lateral and ventral setae of tibiae and metatarsi longer than the diameter of respective segment; preening comb distal in tarsi, very small and plain; metatarsi and tarsi I and II ascopulate, metatarsi and tarsi III and IV densely scopulate but in metatarsi, the length of scopula reaches only 80% of the segment length. Metatarsi I and II with ca. 23, 33 fine ventral setae distally, respectively. *Leg measurements*: Leg I: femur 2.37, patella 2.00, tibia 2.00, metatarsus 2.00, tarsus 1.00, total 9.37. Leg II: 2.25, 1.87, 1.62, 2.12, 1.00, 8.87. Leg III: 2.5, 1.62, 1.5, 2.37, 1.00, 9.00. Leg IV: 3.25, 1.62, 2.00, 2.25, 1.37, 10.5. Formula 4123.

*Trichobothria*: arranged in discontinuous rows; tibiae I–II with 2 rows of 2 in retrodorsal and prodorsal position, respectively; tibiae III with 2 rows of 2 in retrolatero-dorsal and proximo-prodorsal position, respectively; tibiae IV with 2 rows, the first row with 2 in retrolatero-dorsal and the second row with 3 in proximolateral position; metatarsi with 5 in proximo-dorsal position, tarsi I+II with 4 and 6 medio-dorsally, respectively, III+IV with 5 and 6 medio-dorsally, respectively, all trichobothria in medio-dorsal position.

**Figure 4.**
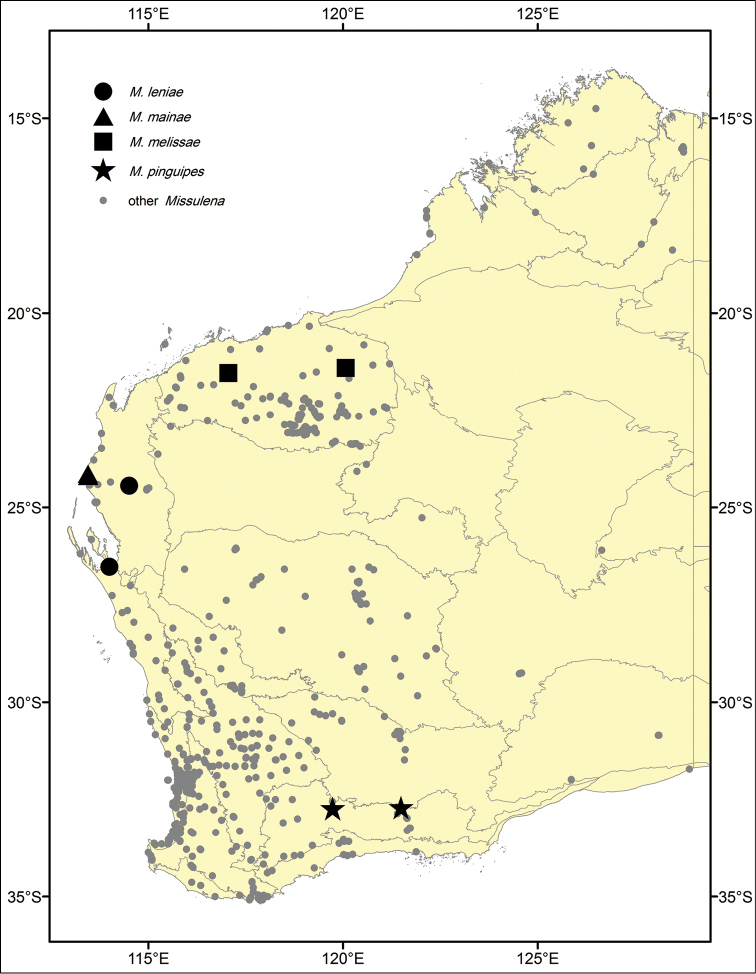
Distribution records of *Missulena* spp. in Western Australia.

*Leg spination*: pedipalp aspinose; leg I: tibia rv0−0−0, v3−3−7, pv1−2−0, d0−0−0; metatarsus rv2−1−1, v2−3−5, pv0−0−0, d0−0−0; tarsus rv1−4−3, v2−7−3, pv2−2−2, d0−0−0; leg II: tibia rv0−0−0, v0−0−0, pv0−1−0, d0−0−0; metatarsus rv0−0−0, v0−0−0, pv0−0−0, d0−0−0; tarsus rv3−5−4, v1−2−2, pv1−3−2, d0−0−0; leg III: tibia rv0−0−0, v0−3−2, pv2−2−2, d2−1−3; metatarsus rv2−2−3, v0−0−0, pv3−3−4, d8−4−2; tarsus rv3−5−4, v0−0−1, pv1−3−4, d0−2−2; leg IV: tibia rv0−2−0, v2−4−4, pv1−1−2, d3−0−0; metatarsus rv1−3−2, v0−0−0, pv1−3−4, d0−0−1; tarsus rv4−9−13, v0−0−1, pv1−4−6, d0−0−2; patellae I and II with ca. 53 and 35 rasps, in 8 and 6 oblique rows prolatero-dorsally, respectively; patella III with ca. 59 rasps widespread in dorsal view (Fig. 4B); patella IV with 19 rasps, in 8 and 6 oblique rows prolatero-dorsally, median rows shorter than lateral rows and with less spines, distal spines forming an interrupted crown of spines in the border of the article (Fig. 4B).

*Tarsal claws*: leg I: 5−4/1; leg II: 6−6/1; leg III: 5−4/1; leg IV: 3−3/1; claws slightly shorter than spines of tarsi.

*Variation in paratypes (N=2)*: total length 6.72−7.36; carapace 3.45 long, 3.90−4.36 wide; number of labial cuspules 14−16, maxillary cuspules 48−67; rastellum with 1−4 thick and conical spines.

###### Distribution.

This species is known from the type locality Millstream-Chichester National Park and Corunna Downs in the Pilbara biogeographic region of Western Australia (Fig. 4).

###### Habitat.

All specimens were collected in pitfall traps. The collecting sites were dominated by *Acacia* spp., with one site having a eucalypt over-storey (McKenzie et al. 2009).

##### Missulena pinguipes sp. n.

http://zoobank.org/4F164298-F7D9-43BC-900F-8B04985F0999

http://species-id.net/wiki/Missulena_pinguipes

**Figure 5.**
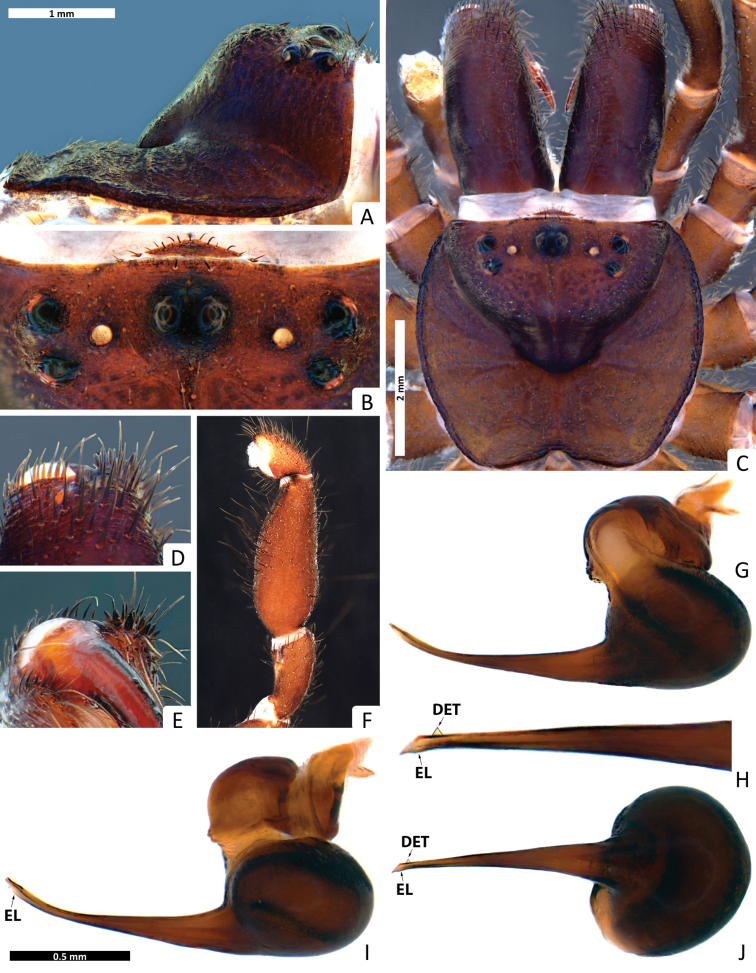
*Missulena pinguipes* sp. n., holotype male (WAM T92331): **A** carapace, lateral view **B** eye group, dorsal view **C** carapace, dorsal view **D** rastellum, dorsal view **E** same, ventral view **F** pedipalp, proventral view **G** bulb and embolus, retrolateral view **H** embolus with apical process depicted, ventral view **I** bulb and embolus, prolateral view **J** same, ventral view. Arrows: (EL) embolar lamella, and (DET) distal embolar tooth.

**Figure 6.**
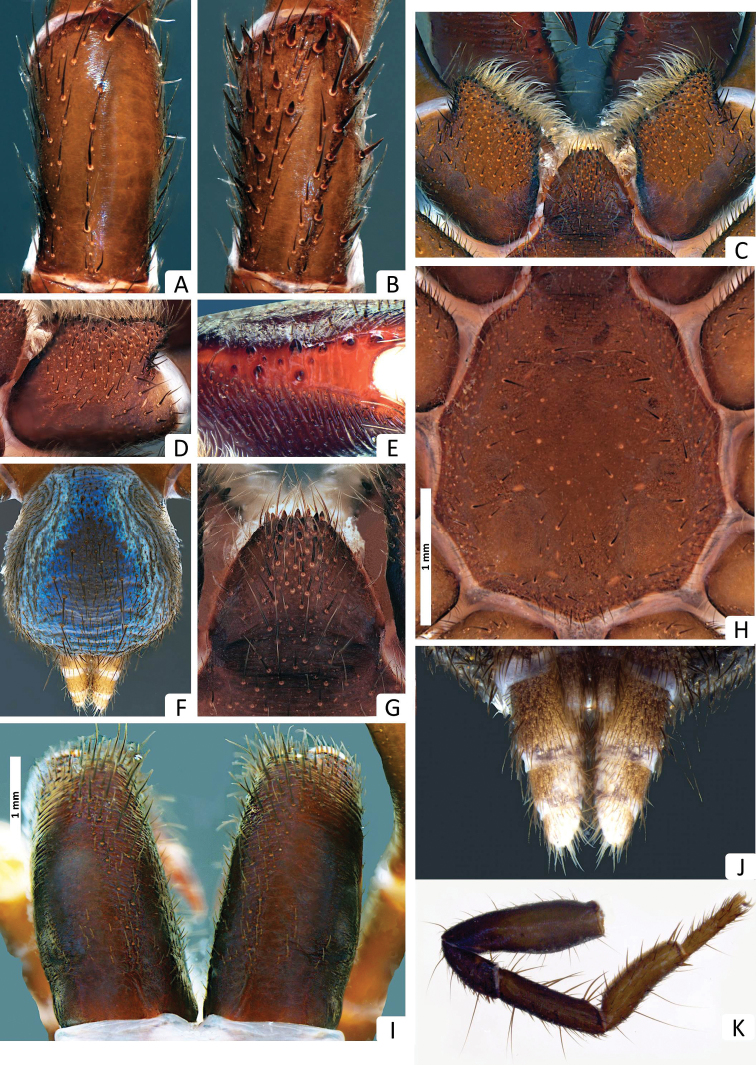
*Missulena pinguipes* sp. n., holotype male (WAM T92331): **A** patella II, dorsal view **B** patella III, dorsal view **C** coxae and labium of pedipalp, ventral view **D** coxae of pedipalp, ventral view **E** cheliceral groove, retroventral view **F** abdomen, dorsal view **G** labium, ventral view **H** sternum and sigillae, ventral view **I** chelicerae, dorsal view **J** spinnerets, ventral view **K** right leg IV, prolateral view.

Figs 1B , 5A–J , 6A–K

###### Type material.

**AUSTRALIA:** ***Western Australia*:** holotype male, Digger Rocks, 89.1 km SE. of Hyden (site DR10), 32°43'58"S, 119°44'03"E, 30 November 2006, dry pitfall trap, D. Kamien (WAM T92331). Paratypes: 4 males, same data as holotype (WAM T92332, T92333, T92334, T92336); 1 male, Exclamation Lake (site SG09B), 32°42'26"S, 121°29'31"E, 23−29 April 2002, dry pitfall trap, R. Teale, G. Harold, A. Sanders and P. Higgs (WAM T45910).

###### Etymology.

The specific epithet is a Latin adjective referring to the swollen metatarsi IV of males (*pinguis*, fat; *pes*, foot).

###### Diagnosis.

Males of *Missulena pinguipes* sp. n. differ from all other named species of the genus, except *Missulena mainae* sp. n. by the swollen metatarsus IV (Fig. 6K). They differ from *Missulena mainae* sp. n. by the brown carapace and chelicerae, which are red in the latter. Females of *Missulena pinguipes* sp. n. are unknown.

###### Description.

Adult male, based on holotype (WAM T92331). Medium-sized mygalomorph spider (total length 5.00).

*Colour*: carapace (Fig. 5A, C) dark reddish-brown, margins dark brown; eye region (Fig. 5B) dark brown, anterior median eyes on black tubercle; chelicerae (Fig. 6I) dark reddish-brown, fangs reddish-brown; abdomen (Fig. 1B, 6F) iridescent blue with light blue longitudinal streaks; sternum (Fig. 6H) yellowish-brown, margins contoured dark yellowish-brown, sigillae yellowish-brown; labium and maxillae dark yellowish-brown (Fig. 6C, G); legs (Fig. 1B) yellowish-brown, tarsi and metatarsi ventrally yellow; spinnerets (Fig. 6J) pale gray, spigots white.

*Carapace*: 2.25 long, 2.37 wide; clypeus 0.17; fovea 0.71; caput and eye region (Fig. 5A) laterally elevated, strongly arched; fovea (Fig. 5C) very deep and strongly procurved, medially extending as triangular depression (Fig. 5C), pars cephalica with few granulations around the eyes and between the eyes and fovea, pars thoracica rugose with bands of fine, random fissures centered around fovea (Fig. 5C).

*Eyes*: OQ 3.5 times wider than long, occupying 0.83 of cephalic width; OAW 1.97; OAL 1.42; IPF 0.74; width of anterior eye group 1.35, with of posterior group 1.17, OQ length 0.38; PME 0.1; PLE 0.1; ALE 0.08; AME 0.1, AME on tubercle, 0.31 long, 0.41 wide; AME inter-distance 0.1; AME to ALE 0.47; AME to PME 0.11; PLE to ALE 0.22; PLE to PME 0.2; PME inter-distance 0.65; PME to ALE 0.27; eye region (Fig. 5B) with reduced setation although some setae present anterior to AME, between lateral eyes and between posterior eyes and fovea.

*Chelicerae*: 1.57 long, 0.82 wide; distally broad, diagonal, slightly conical; edges smoothly rounded; with weak transverse ridges which distally extend over entire length (Fig. 6I), without setae in area of transverse ridges but with ca. 30 setae along inner margin of chelicera; rastellum developed, pronounced, consisting of a sclerotised process with 9 strong conical spines and 18−22 disordered setae (Fig. 5D), 9 long setae extend forward from anterior margin of each chelicera and cover base of fang, setae largest on latero-ventral side; inner margin of cheliceral furrow with 3 rows of teeth (Fig. 6E); prolateral (inner) row with ca. 6 teeth, all teeth spaced; intermediate row with 3 proximal, spaced teeth; retrolateral (outer) row with 3 proximal, spaced teeth; with 1 distal tooth.

*Maxillae*: 0.91 long; 0.71 wide, almost square (Fig. 6C, D), ca. 64 pointed cuspules along entire anterior margin, distally pointed and extended into a prominent heel.

*Labium*: 0.51 long, 0.45 wide; conical, ca. 17 pointed cuspules anteriorly (Fig. 6G); labiosternal suture developed as a shallow groove; a pair of sigilla near labiosternal suture (Fig. 6H), developed as irregular, poorly-defined patches.

*Sternum*: 1.37 long, 1.48 wide; oval and rebordered (Fig. 6H), with prominent setae, arranged irregularly but denser lateral to labium; 4 pairs of sigillae, anterior and second pair (anterior-posterior) smallest but well defined, third pair bigger than 2 anterior pairs and well defined, and posterior pair bigger than all others, roughly oval but not well defined, all sigillae slightly depressed.

*Abdomen*: 2.28 long, 2.00 wide; roughly oval (Fig. 6F); 4 spinnerets (Fig. 6J), PLS 0.85 long, 0.37 wide, apical segment domed; PMS 0.31 long, 0.14 wide.

*Pedipalp*: length of trochanter 0.74, femur 1.62, patella 0.92, tibia 1.51, tarsus 0.55; entire palp is aspinose, femur longer than tibia, tarsus terminally blunt (Fig. 5F); bulb pyriform and rather stout than globular (Fig. 5G−J), 2 strongly sclerotised sections connected by a velar median structure (“haematodocha”); bulb strongly twisted proventrally (Fig. 5G, I); embolus short, tapering and slightly twisted medially (Fig. 5H, J); embolus tip triangular, with a lamella well-developed (EL) and a very small tooth (DET) in ventral view (Fig. 5H, J).

*Legs*: with few brown setae, ventral setae of tibiae and metatarsi generally much longer and thicker than dorsal setae and bent towards the exterior; dorsal, lateral and ventral setae of tibiae and metatarsi longer than the diameter of respective segment; preening comb distal in tarsi, very small and plain; metatarsi I, II and III ascopulate; metatarsus IV swollen with dense scopula ventrally across entire length (Fig. 6K); tarsi I, II, III and IV ascopulate but with ca. 33, 24, 21, 17 fine ventral setae distally, respectively. *Leg measurements*: Leg I: femur 2.11, patella 1.03, tibia 1.51, metatarsus 1.44, tarsus 0.85, total 6.96. Leg II: 1.81, 0.96, 1.07, 1.33, 0.85, 6.03. Leg III: 1.74, 0.92, 1.14, 1.22, 0.77, 5.81. Leg IV: 1.85, 1.00, 1.25, 1.29, 0.74, 6.14. Formula 4123.

*Trichobothria*: arranged in discontinuous rows; tibiae I–II with 1 row of 3 in retrolateral and dorsal position, respectively; tibiae III-IV with 2 rows of 4−5 in dorsal position, first row situated prodorsally and second row situated retrodorsally; metatarsi with 2 in medio-dorsal position, tarsi I+II with 2, III+IV with 3, all trichobothria in medio-dorsal position.

*Leg spination*: pedipalp aspinose; leg I: tibia rv0−0−0, v3−3−7, pv0−0−0, d0−0−0; metatarsus rv2−1−1, v2−3−5, pv0−0−0, d0−0−0; tarsus rv1−1−1, v1−3−2, pv0−0−0, d0−0−0; leg II: tibia rv0−0−0, v3−3−4, pv0−0−0, d0−0−0; metatarsus rv0−1−0, v3−2−3, pv0−1−0, d0−0−0; tarsus rv0−2−0, v0−3−0, pv0−2−0, d0−0−0; leg III: tibia rv1−1−1, v2−2−5, pv0−0−1, d0−0−4; metatarsus rv0−2−0, v2−5−4, pv0−1−0, d0−0−3; tarsus rv0−2−1, v1−3−3, pv0−0−1, d0−0−2; leg IV: tibia rv0−0−0, v3−5−4, pv0, d1−0−2; metatarsus rv2−3−1, v0−0−0, pv3−4−3, d0−1−2; tarsus rv1−2−1, v1−5−3, pv0, d0−0−2; patellae I, II without rasps and spines (Fig. 6A), patella III with ca. 26 rasps in 8 oblique rows dorsally, median rows shorter than lateral rows and with less spines, distal spines forming a interrupted crown of spines in the border of the article (Fig. 6B); patella IV with 6 rasps retrolaterally and 12 thick and short spines dorsally.

*Tarsal claws*: leg I: 3−2/2; leg II: 3−4/2; leg III: 4−3/2; leg IV: 1−2/ 0−1; claws slightly shorter than spines of tarsi.

*Variation in paratypes (N=5)*: total length 4.00−5.00; carapace 1.77−2.37 long, 2.37−2.6 wide; number of labial cuspules 15−30, maxillary cuspules 40−66; rastellum with 6–11 thick and conical spines.

###### Distribution.

This species is known only from the Mallee biogeographic region of southern Western Australia (Fig. 4).

###### Phenology and habitat preferences.

The specimens were collected in pitfall traps in woodland habitats in either April or November.

##### Missulena leniae sp. n.

http://zoobank.org/BB6C03F8-C9BF-4F1E-A92C-2A7699408800

http://species-id.net/wiki/Missulena_leniae

**Figure 7.**
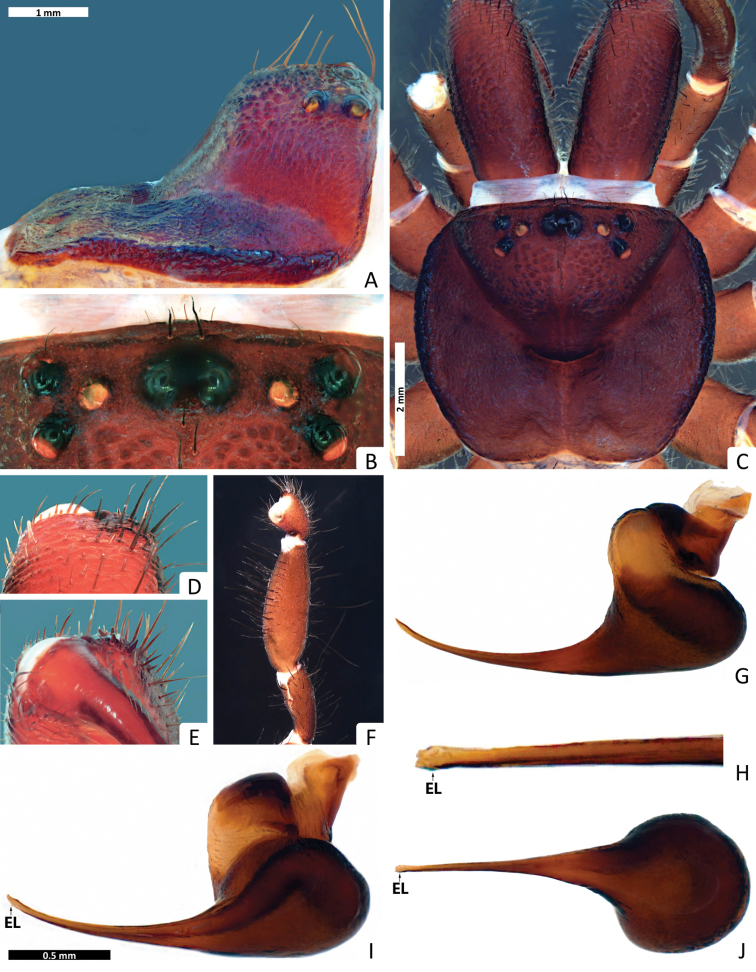
*Missulena leniae* sp. n., holotype male (WAM T96784): **A** carapace, lateral view **B** eye group, dorsal view **C** carapace, dorsal view **D** rastellum, dorsal view **E** same, ventral view **F** pedipalp, proventral view **G** bulb and embolus, retrolateral view **H** embolus, ventral view **I** bulb and embolus, prolateral view **J** same, ventral view. Arrows: (EL) embolar lamella.

**Figure 8.**
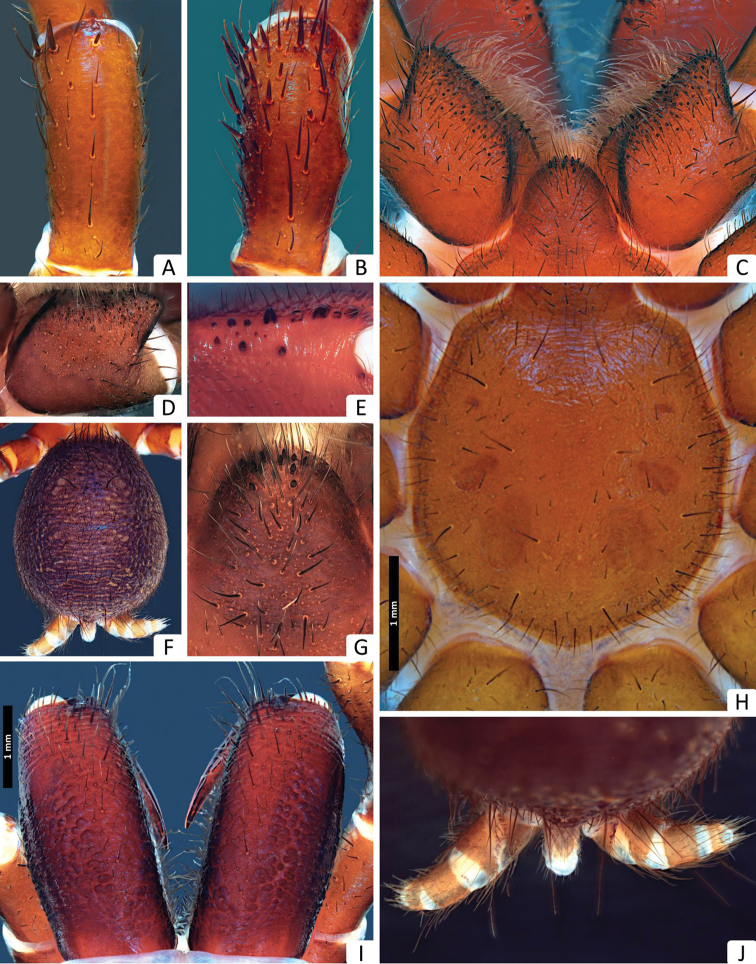
*Missulena leniae* sp. n., holotype male (WAM T96784): **A** patella II, dorsal view **B** patella III, dorsal view **C** coxae and labium of pedipalp, ventral view **D** coxae of pedipalp, ventral view **E** cheliceral groove, retroventral view **F** abdomen, dorsal view **G** labium, ventral view **H** sternum and sigillae, ventral view **I** chelicerae, dorsal view **J** spinnerets, dorsal view.

Figs 1C , 7A–J , 8A–J

Missulena sp. 4: Main et al. 2000: 285.

###### Type material.

**AUSTRALIA:** ***Western Australia*:** holotype male, Nanga Station, site NA3, 26°31'20.9"S, 114°00'08.3"E, 12 May−3 August 1995, pitfall trap, N. Hall (WAM T96784). Paratype: 1 male, Mardathuna Station, site MR2, 24°26'35.7"S, 114°30'41.5"E, 25 May−26 August 1995, pitfall trap, N. Hall (WAM T96785).

###### Etymology.

The specific epithet is a patronym in honour of the second author’s daughter, Leni Elise Harms.

###### Diagnosis.

Males of *Missulena leniae* sp. n. differ from other small species (carapace < 4 mm) without red colouration on chelicerae and carapace by the weak rastellum without conical spines (elevated and with conical spines in *Missulena faulderi*, *Missulena melissae* and *Missulena rutraspina*), the presence of granulations on carapace and chelicerae (except *Missulena pinguipes* and *Missulena torbayensis*), the presence of long setae on the chelicerae (absent in *Missulena faulderi*, *Missulena melissae* and *Missulena rutraspina*), patella III with rasps (all patellae in *Missulena torbayensis*), pars cephalica dark brown (black in *Missulena dipsaca*) and abdomen with metallic blue lines (lacking in *Missulena dipsaca*). Females of *Missulena leniae* sp. n. are unknown.

###### Description.

Adult male, based on holotype (WAM T96784). Medium-sized mygalomorph spider (total length 6.81).

*Colour*: carapace (Figs 1C, 7A, C) dark reddish-brown, margins dark brown; eye region (Fig. 7B) dark reddish-brown, anterior median eyes on black tubercle; chelicerae (Fig. 8I) dark reddish-brown, fangs reddish-brown; abdomen (Fig. 8F) pale grey with few light blue longitudinal streaks; sternum (Fig. 8H) yellowish-brown, margins contoured dark yellowish-brown, sigillae yellowish-brown; labium and maxillae dark red-yellowish-brown (Fig. 8C, G); legs (Fig. 1C) yellowish-brown, tarsi and metatarsi ventrally pale yellowish-brown; spinnerets (Fig. 8J) pale gray, spigots white.

*Carapace*: 2.63 long, 3.18 wide; clypeus 0.15; fovea 1.06; caput and eye region (Fig. 7B) laterally elevated, strongly arched; fovea (Fig. 7C) very deep and strongly procurved, medially extending as a very deep triangular depression; carapace with numerous large granulations, widespread over carapace and dorsally on chelicerae; weaker on pars thoracica, with bands of fine, random fissures centered around fovea (Fig. 7C).

*Eyes*: OQ 2.89 times wider than long, occupying 1.34 of cephalic width; OAW 2.4; OAL 1.62; IPF 0.91; width of anterior eye group 1.57, with of posterior group 1.45, OQ length 0.54; PME 0.14; PLE 0.14; ALE 0.20; AME 0.16, AME on tubercle, 0.31 long, 0.59 wide; AME inter-distance 0.11; AME to ALE 0.40; AME to PME 0.14; PLE to ALE 0.20; PLE to PME 0.25; PME inter-distance 0.77; PME to ALE 0.22; eye region (Fig. 7B) with reduced setation although some setae present anterior to AME, anterior ALE and between posterior eyes and fovea.

*Chelicerae*: 2.04 long, 0.95 wide; distally broad, diagonal, slightly conical; edges smoothly rounded; with transverse ridges which distally extend over entire length and many strong granulations widespread in dorsal view (Fig. 8I), with ca. 22 long setae widespread in inner area of transverse ridges and with more than 70 short setae along inner margin of chelicera; rastellum (Fig. 7D) poorly developed, weakly pronounced, consisting of a sclerotised process with 2 strong conical spines and 9−10 disordered setae, 9 long setae extend forward from anterior margin of each chelicera and cover base of fang, setae largest on latero-ventral side; inner margin of cheliceral furrow with 3 rows of teeth (Fig. 8E); prolateral (inner) row with ca. 9 teeth, all teeth spaced; intermediate row with 4 proximal, spaced teeth; retrolateral (outer) row with 3 proximal, spaced teeth.

*Maxillae*: 1.25 long; 0.93 wide, longer than wide (Fig. 8C, D), ca. 86 pointed cuspules along entire anterior margin, distally pointed and extended into a prominent heel.

*Labium*: 0.63 long, 0.61 wide; conical, ca. 12 pointed cuspules anteriorly (Fig. 8G); labiosternal suture developed as a shallow groove; a pair of sigilla near labiosternal suture (Fig. 8H), developed as irregular, very small and poorly-defined patches.

*Sternum*: 1.95 long, 1.72 wide; oval and rebordered (Fig. 8H), with prominent setae, arranged irregularly but denser lateral to labium; 4 pairs of sigillae, anterior pair very small, irregular and undefined; second pair (anterior-posterior) smallest but well defined; third pair bigger than 2 anterior pairs and well defined; and posterior pair bigger than all others, roughly oval but not well defined; 3 posterior sigillae slightly depressed.

*Abdomen*: 3.31 long, 3.04 wide; roughly oval (Fig. 8F); 4 spinnerets (Fig. 8J), PLS 1.22 long, 0.40 wide, apical segment domed; PMS 0.29 long, 0.15 wide.

*Pedipalp*: length of trochanter 0.75, femur 2.54, patella 1.09, tibia 1.90, tarsus 0.81; entire palp is aspinose, femur longer than tibia, tarsus terminally blunt (Fig. 7F); bulb pyriform and rather stout than globular (Fig. 7G, I−J), 2 strongly sclerotised sections connected by a velar median structure (“haematodocha”); bulb strongly twisted proventrally (Fig. 7G, I); embolus short, strong, tapering and slightly twisted medially (Fig. 7H, J); embolus tip triangular in prolateral/retrolateral views and subquadrate in ventral view, with a small lamella (EL) and without tooth in ventral view, duct straight in proximal embolus (Fig. 7H, I).

*Legs*: with few brown setae, ventral setae of tibiae and metatarsi generally much longer and thicker than dorsal setae and bent towards the exterior; dorsal, lateral and ventral setae of tibiae and metatarsi longer than the diameter of respective segment; preening comb distal in tarsi, very small and plain; metatarsi I and II ascopulate; metatarsi III and IV with a weak scopula occupying 75% of segment length; tarsi I and II ascopulate, tarsi III and IV with a weak scopula along entire length; metatarsi I and II with ca. 57 and 47 fine ventral setae distally, respectively. *Leg measurements*: Leg I: femur 3.14, patella 1.33, tibia 1.81, metatarsus 2, tarsus 1.22, total 9.51. Leg II: 2.66, 1.33, 1.77, 1.88, 1.22, 8.88. Leg III: 2.85, 1.40, 1.74, 1.96, 1.29, 9.25. Leg IV: 3.14, 1.33, 2.03, 2.03, 1.37, 9.92. Formula 4123.

*Trichobothria*: arranged in discontinuous rows; tibiae I–III with 2 rows of 2 in prodorsal position and 3 in retrodorsal position, respectively; tibiae IV with 7 widespread in dorsal position; metatarsi with 3 in proximo-dorsal position; tarsi I-IV with 3, all trichobothria in a row in medio-dorsal position.

*Leg spination*: pedipalp aspinose; leg I: tibia rv0−0−2, v2−3−3, pv0−0−0, d0−0−0; metatarsus rv0−2−1, v2−2−3, pv0−0−0, d0−0−0; tarsus rv0−1−1, v2−4−3, pv0−0−0, d0−0−0; leg II: tibia rv0−0−2, v2−3−3, pv0−0−0, d0−0−0; metatarsus rv0−1−2, v0−4−3, pv0−0−0, d0−0−0; tarsus rv0−2−0, v3−2−4, pv0−0−0, d0−0−0; leg III: tibia rv0−0−3, v0−2−7, pv0−0−2, d2−0−10; metatarsus rv1−1−2, v0−0−0, pv1−1−1, d1−1−3; tarsus rv1−3−2, v0−1−2, pv0−0−0, d0−1−2; leg IV: tibia rv0−0−0, v3−3−4, pv0−0−1, d1−1−2; metatarsus rv1−1−2, v0−0−0, pv1−2−3, d0−0−0; tarsus rv2−6−6, v1−3−2, pv0−0−0, d0−0−2; patellae I with ca. 7 rasps in 3 proximal oblique rows dorsally, patellae II with 1 rasp (Fig. 8A), patella III with ca. 16 rasps and 4 spines in 8 oblique rows dorsally, median rows shorter than lateral rows and with less rasps/spines, distal rasps/spines forming a interrupted crown of rasps/spines in the border of the article (Fig. 8B); patella IV with 4 rasps retrolaterally and 8 thick and short spines prodorsally.

*Tarsal claws*: leg I: 6−5/3; leg II: 4−5/3; leg III: 3−2/1; leg IV: 2−2/1; claws slightly shorter than spines of tarsi.

*Variation in paratype (N=1)*: total length 4.90; carapace 2.63 long, 2.54 wide; number of labial cuspules 58−70, maxillary cuspules 14; rastellum with 4−4 thick and conical spines.

###### Distribution.

This species is currently known from two sites located in the Carnarvon and Yalgoo biogeographic regions of Western Australia (Fig. 4).

###### Phenology and habitat preferences.

The two specimens were collected in pitfall traps between May and August. They were listed as *Missulena* sp. 4 in a survey of mygalomorph spiders of the southern Carnarvon Basin by Main et al. (2000). The two sites are dominated by *Banksia* and eucalypt mallee woodland (site NA3), or *Acacia aneura* (site MR2) over stable but sandy substrates (Burbidge et al. 2000, Appendix A; Wyrwoll et al. 2000).

##### Missulena mainae sp. n.

http://zoobank.org/FA26CB3C-43A2-4DA7-AFD9-AC84E3366B1D

http://species-id.net/wiki/Missulena_mainae

**Figure 9.**
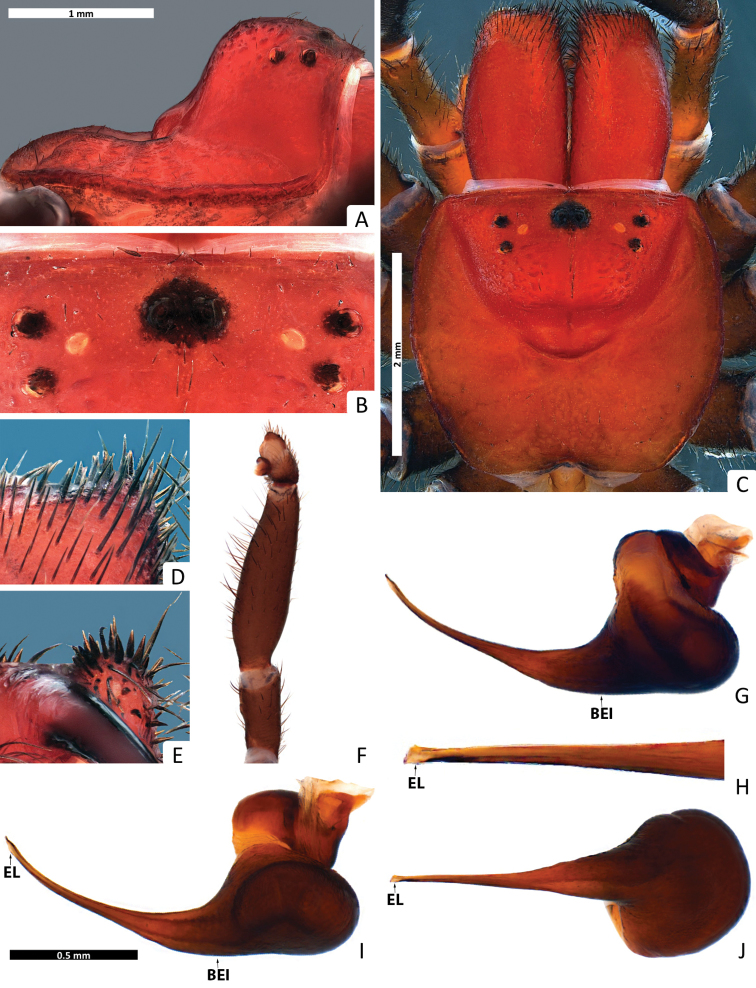
*Missulena mainae* sp. n., holotype male (WAM T96782): **A** carapace, lateral view **B** eye group, dorsal view **C** carapace, dorsal view **D** rastellum, dorsal view **E** same, ventral view **F** pedipalp, proventral view **G** bulb and embolus, retrolateral view **H** embolus, ventral view **I** bulb and embolus, prolateral view **J** same, ventral view. Arrows: (EL) embolar lamella, and (BEI) basal embolar intumescence.

**Figure 10.**
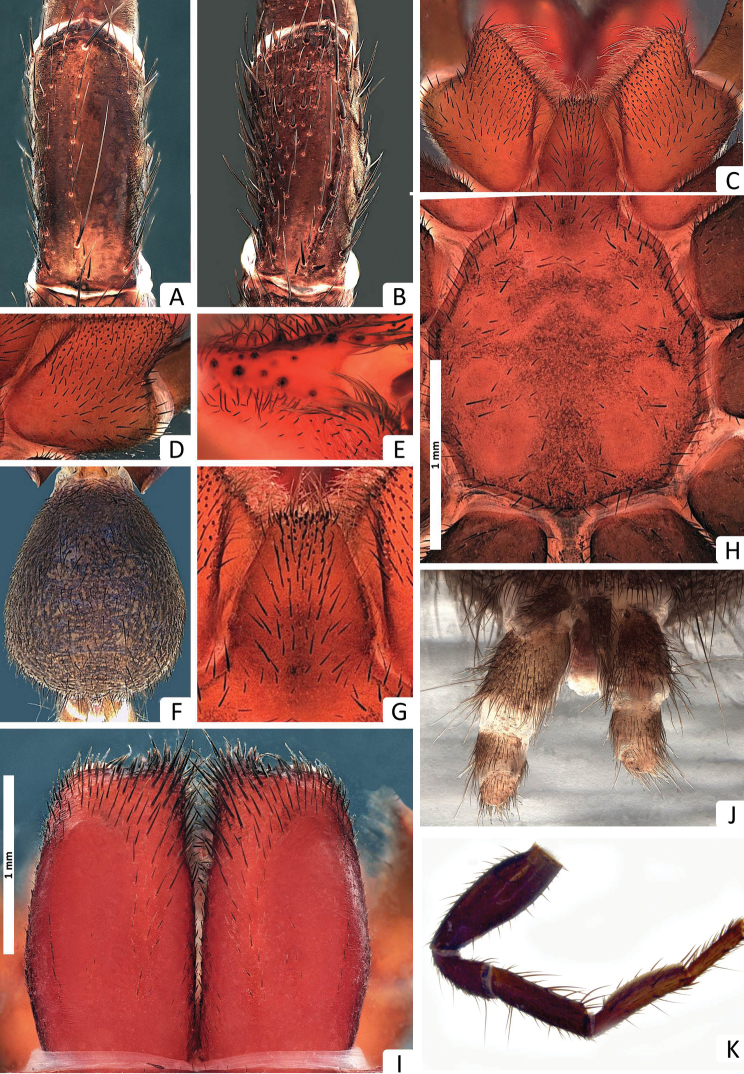
*Missulena mainae* sp. n., holotype male (WAM T96782): **A** patella II, dorsal view **B** patella III, dorsal view **C** coxae and labium of pedipalp, ventral view **D** coxae of pedipalp, ventral view **E** cheliceral groove, retroventral view **F** abdomen, dorsal view **G** labium, ventral view **H** sternum and sigillae, ventral view **I** chelicerae, dorsal view **J** spinnerets, ventral view **K** right leg IV, prolateral view.

Figs 1D , 9A–J , 10A–K

Missulena sp. 2: Main et al. 2000: 285.

###### Type material.

**AUSTRALIA:** ***Western Australia*:** holotype male, Cape Cuvier, Quobba Station, site CU6, 24°08'20.4"S, 113°26'43.9"E, 31 May−25 August 1995, pitfall trap (WAM T96782). Paratypes: 2 males, Cape Cuvier, Quobba Station, site CU5, 24°11'34.0"S, 113°27'17.4"E, 27 September−2 October 1994, dry pitfall trap, P. West et al. (WAM T96781); 3 males, same data, 29 May−25 August 1995, N. Hall (WAM T96783).

###### Etymology.

This species is named in honour of Barbara York Main in recognition of her substantial contributions to arachnology. She also was the first to recognize this taxon as a distinctive new species (Main et al. 2000).

###### Diagnosis.

Males of *Missulena mainae* sp. n. differ from all other species by the uniformly red dorsal coloration of the carapace (pars cephalica and thoracica red; Figs 2D, 10C). Males share with *Missulena pinguipes* sp. n. the presence of a swollen metatarsus IV, but the character is less pronounced in *Missulena mainae* (Fig. 10K).

###### Description.

Adult male, based on holotype (WAM T96782). Medium-sized mygalomorph spider (total length 7.90).

*Colour*: carapace (Fig. 9C) pale red, margins pale red; eye region (Fig. 9B) pale red, anterior median eyes on black tubercle; chelicerae (Fig. 10I) pale red, fangs dark red; abdomen (Fig. 10F) pale grey with little spots of blue and light gray longitudinal streaks; sternum (Fig. 10H) pale red, margins contoured pale brown, sigillae pale red and spots pale brown; labium (Fig. 10G) and maxillae pale red; legs (Fig. 1D) brown, coxae and trochanter pale brown; spinnerets (Fig. 10J) pale gray, spigots white.

*Carapace*: 2.59 long, 2.77 wide; clypeus 0.09; fovea 0.75; caput and eye region (Fig. 9A) laterally elevated, strongly arched in a rectangular form; fovea (Fig. 9C) very deep and strongly procurved, medially extending as triangular depression, pars cephalica with few granulations around the eyes, behind the eyes and between the eyes and fovea, pars thoracica with bands of fine, random fissures centered around fovea (Fig. 9C).

*Eyes*: OQ 3.06 times wider than long, occupying 1.26 of cephalic width; OAW 2.20; OAL 1.65; IPF 0.80; width of anterior eye group 1.40, with of posterior group 1.25, OQ length 0.45; PME 0.10; PLE 0.13; ALE 0.11; AME 0.16, AME on tubercle, 0.24 long, 0.42 wide; AME inter-distance 0.07; AME to ALE 0.44; AME to PME 0.22; PLE to ALE 0.17; PLE to PME 0.13; PME inter-distance 0.79; PME to ALE 0.16; eye region (Fig. 9B) with reduced setation although some setae present anterior to AME, between lateral eyes and between posterior eyes and fovea.

*Chelicerae*: 1.42 long, 0.88 wide; distally broad, diagonal, slightly conical; edges smoothly rounded; without transverse ridges (Fig. 10I), with 2 lines of dorsal setae, prolateral with ca. 28 and retrolateral with ca. 15; with ca. 30 setae along inner margin of chelicera; rastellum (Fig. 9D, E) developed, pronounced, consisting of a sclerotised process with 11 strong conical spines and 16 disordered setae, 13 long setae extend forward from anterior margin of each chelicera and cover base of fang, setae largest on latero-ventral side; inner margin of cheliceral furrow with 3 rows of teeth (Fig. 10E); prolateral (inner) row with ca. 6 teeth, all teeth spaced; intermediate row with 5 proximal, spaced teeth; retrolateral (outer) row with 4 proximal, spaced teeth; with 2 distal teeth.

*Maxillae*: 1.31 long; 1.0 wide, longer than wide, almost square (Fig. 10C, D), ca. 96 pointed cuspules along entire anterior margin, distally pointed and extended into a prominent heel.

*Labium*: 0.80 long, 0.62 wide; conical, ca. 31 pointed cuspules anteriorly (Fig. 10G); labiosternal suture developed as a shallow groove; a pair of sigilla near labiosternal suture (Fig. 10H), developed as irregular, poorly-defined patches.

*Sternum*: 1.82 long, 1.71 wide; oval and rebordered (Fig. 10H), with prominent setae, arranged irregularly but denser lateral to labium; 4 pairs of sigillae, anterior pair smallest than others and undefined, second pair (anterior-posterior) smallest but well defined, third pair bigger than 2 anterior pairs and well defined, and posterior pair bigger than all others, roughly oval but not well defined, all sigillae slightly depressed.

*Abdomen*: 3.31 long, 2.54 wide; roughly oval (Fig. 10F); 4 spinnerets (Fig. 10J), PLS 1.00 long, 0.40 wide, apical segment domed; PMS 0.34 long, 0.15 wide.

*Pedipalp*: length of trochanter 1.0, femur 2.27, patella 1.0, tibia 2.09, tarsus 0.63; entire palp is aspinose, femur longer than tibia, tarsus terminally blunt (Fig. 9F); bulb pyriform and rather stout than globular (Fig. 9G−J), 2 strongly sclerotised sections connected by a velar median structure (“haematodocha”); bulb strongly twisted proventrally (Fig. 9G, I); embolus short, with a proximal intumescence (BEI) in prolateral view, tapering and slightly twisted medially (Fig. 9G−J); embolus tip triangular in prolateral/retrolateral view and rectangular in ventral view, with a small lamella (EL) and without tooth (Fig. 9H).

*Legs*: with few brown setae, ventral setae of tibiae and metatarsi generally much longer and thicker than dorsal setae and bent towards the exterior; dorsal, lateral and ventral setae of tibiae and metatarsi longer than the diameter of respective segment; preening comb distal in tarsi, very small and plain; metatarsi I, II and III ascopulate, tarsi I, II, III and IV ascopulate but with ca. 20, 24, 17, 13 fine ventral setae distally, respectively; metatarsus IV with dense scopula ventrally across entire length. *Leg measurements*: Leg I: femur 1.55, patella 1.22, tibia 1.40, metatarsus 1.48, tarsus 0.88, total 6.55. Leg II: 2.22, 1.18, 1.44, 1.48, 1, 7.33. Leg III: 2.18, 1.18, 1.48, 1.48, 1.03, 7.37. Leg IV: 2.22, 1.29, 1.55, 1.55, 1.03, 7.66. Formula 4123.

*Trichobothria*: arranged in discontinuous rows; tibiae I–II with 2 proximal rows of 3 in retrodorsal and prodorsal position, respectively; tibiae III-IV with 2 rows of 2−3 in prodorsal/retrolateral position; metatarsi with 4 in mediodorsal position, tarsi I+II with 2, III+IV with 3, all trichobothria in mediodorsal position.

*Leg spination*: pedipalp aspinose; leg I: tibia rv1−0−1, v2−3−5, pv0−0−0, d0−0−0; metatarsus rv0−0−1, v2−6−5, pv1−1−2, d0−0−0; tarsus rv0−0−0, v3−6−7, pv0−0−0, d0−0−0; leg II: tibia rv1−0−0, v3−4−4, pv0−0−0, d0−0−0; metatarsus rv1−2−1, v2−5−4, pv1−1−2, d0−0−0; tarsus rv0−1−1, v3−4−8, pv0−1−0, d0−0−0; leg III: tibia rv0−0−0, v1−2−3, pv0−0−1, d0−1−7; metatarsus rv1−2−2, v4−6−8, pv1−0−1, d3−3−3; tarsus rv0−0−0, v1−4−4, pv0−0−1, d0−0−3; leg IV: tibia rv0−0−0, v1−2−2, pv0−0−1, d1−0−4; metatarsus rv1−2−2, v0−0−0, pv1−4−3, d2−1−3; tarsus rv1−1−3, v3−4−6, pv0−0−0, d0−0−2; patellae I, II without rasps and spines (Fig. 10A), patella III with ca. 28 rasps in 8 oblique rows dorsally, median rows shorter than lateral rows and with less spines, distal spines forming a interrupted crown of spines in the border of the article (Fig. 10B); patella IV with 12 rasps retrolaterally and 6 thick and short spines dorsally.

*Tarsal claws*: leg I: 3−2/2; leg II: 6−6/2; leg III: 6−5/3; leg IV: 5−5/3; claws slightly shorter than spines of tarsi.

*Variation in paratypes (N=5)*: total length 5.27−6.09; carapace 2.36−2.63 long, 2.54−3.27 wide; number of labial cuspules 19−26, maxillary cuspules 86−108; rastellum with 10–13 thick and conical spines.

###### Distribution.

This species is known only from Quobba Station in the Carnarvon biogeographic region of Western Australia (Fig. 4).

###### Phenology and habitat preferences.

All specimens were collected in pitfall traps in a period between May and October. They were listed as *Missulena* sp. 2 in a survey of mygalomorph spiders of the southern Carnarvon Basin (Main et al. 2000). The two sites are dominated by *Acacia* over dune substrates (Burbidge et al. 2000, Appendix A; Wyrwoll et al. 2000).

##### Key to the described males of *Missulena* from Australia

(Distribution indicative as in Table 1; some species may have a wider occurrence.)

**Table:** 

1	Chelicerae, and sometimes parts of the carapace with red markings	2
−	Chelicerae and carapace brown or black	6
2	Pars cephalica *and* thoracica uniformly red [WA: Carnarvon]	*Missulena mainae* sp. n.
−	Pars cephalica red but pars thoracica black or brown	3
3	Pars cephalica uniformly red	4
−	Pars cephalica almost black but with traces of dark-red [WA: Southwest]	*Missulena hoggi*^1^
4	Small species (carapace length < 3.0 mm); rastellum with fewer than 6 spines [WA: Pilbara]	*Missulena langlandsi*
−	Large species (carapace length > 4.0 cm); rastellum with more than 6 spines	5
5	Abdomen dark yellow but with a lighter patch anteriorly [SA]	*Missulena reflexa*
−	Abdomen dark brown and without yellow patch	*Missulena occatoria* [NSW, Qld, Vic, ACT, SA, NT, WA] and *Missulena insignis* [WA]^2^
6	Abdomen dorsally with pale colouration	7
–	Abdomen dorsally lacks pale colouration, mainly brownish-grey or metallic blue	8
7	Abdomen dorsally greyish-white; rastellum with 10 spines [WA: Kimberleys; NT; Qld]	*Missulena pruinosa*
−	Abdomen dorsally with a bluish-grey patch in anterior position but otherwise dark brown; rastellum with 5–6 spines [Qld, NSW, Vic]	*Missulena bradleyi*
8	Outer surface of chelicerae with longitudinal ridges	9
–	Outer surface of chelicerae smooth	11
9	Patella II prolaterally with rasps; tip of embolus with process [WA: Pilbara]	*Missulena melissae* sp. n.
−	Patella II without rasps; tip of embolus without processes	10
10	Proventral teeth of cheliceral furrow fused; embolus significantly longer than bulb and medially curved; patella III with fewer than 40 rasps [WA: Pilbara]	*Missulena faulderi*
−	Proventral teeth of cheliceral furrow not fused; embolus not significantly longer than bulb and straight; patella III with greater than 50 rasps [WA, SA, Vic]	*Missulena rutraspina*
11	Length of carapace > 3.0 mm	12
−	Length of carapace < 3.0 mm	14
12	Rasps present on patella I	13
−	Rasps absent on patella I; tarsus I ascopulate; thin scopula on tarsus III; sternum without pair of sigilla in labial groove [NSW, Vic, SA, WA, Qld]	*Missulena dipsaca*
13	Length of carapace < 4.0 mm, rasps on all patellae [WA: Southwest]	*Missulena torbayensis*
−	Length of carapace > 5.0 mm, rasps on patellae I and III only [WA: Southwest]	*Missulena granulosa*
14	Abdomen entirely metallic blue, patella III with more than 25 rasps [WA: Mallee]	*Missulena pinguipes* sp. n.
−	Abdomen with some metallic blue markings but otherwise reddish brown; patella III with fewer than 25 rasps [WA: Carnarvon and Yalgoo]	*Missulena leniae* sp. n.

^1^ Faulder (1995a), in an unpublished thesis, considers *Missulena hoggi* and *Missulena granulosa*, mainly differentiated by the colour pattern of carapace and chelicerae, conspecific.

^2^ There is confusion about the identity of *Missulena insignis* and *Missulena occatoria*. Both species cannot be diagnosed based on the original description. The holotype of *Missulena insignis* is from Swan River, Western Australia and that of *Missulena occatoria* from and unidentified locality in “New Holland” (= Australia). Main (1985) suggested referring Western Australian specimens to *Missulena insignis* (the ‘western’ species) and eastern Australian specimens to *Missulena occatoria* (the ‘eastern’ species).

## Supplementary Material

XML Treatment for Missulena melissae

XML Treatment for Missulena pinguipes

XML Treatment for Missulena leniae

XML Treatment for Missulena mainae

## References

[B1] Brunet B (1994) The silken web: a natural history of Australian spiders. Reed, Chatswood, N.S.W., 208 pp.

[B2] Brunet B (2000) Spider watch: a guide to Australian spiders. New Holland, Chatswood, N.S.W., 177 pp.

[B3] Burbidge AH Harvey MS McKenzie NL (Eds) (2000) Biodiversity of the southern Carnarvon Basin. Records of the Western Australian Museum, Supplement 61: 595 pp

[B4] Department of the Environment (2013) Australia’s bioregions (IBRA). http://www.environment.gov.au/topics/land/national-reserve-system/science-maps-and-data/australias-bioregions-ibra [accessed 28 October 2013]

[B5] Durrant BJ Harvey MS Framenau VW Ott R Waldock JM (2010) Patterns in the composition of ground-dwelling spider communities in the Pilbara bioregion, Western Australia. Records of the Western Australian Museum, Supplement 78: 185-204

[B6] Faulder RJ (1995a) Systematics and Biogeography of the Spider Genus *Missulena* Walckenaer. Master of Science thesis, University of Sydney, Sydney

[B7] Faulder RJ (1995b) Two new species of the Australian spider genus *Missulena* Walckenaer (Aranenae: Actinopodidae). Records of the Western Australian Museum Supplement 52: 73-78

[B8] Goloboff PA Platnick NI (1987) A review of the Chilean spiders of the superfamily Migoidea (Araneae, Mygalomorphae). American Museum Novitates 2888: 1-15

[B9] Griswold CE Ledford J (2001) A monograph of the migid trap door spiders of Madagascar and review of the world genera (Araneae, Mygalomorphae, Migidae). Occasional Papers of the California Academy of Sciences 151: 1-120

[B10] Harms D Framenau VW (2013) New species of mouse spiders (Araneae: Mygalomorphae: Actinopodidae: *Missulena*) from the Pilbara region, Western Australia. Zootaxa 3637: 521-540 26046218

[B11] Herzig V Khalife AA Youmie C Isbister GK Currie BJ Churchill TB Horner S Escoubas P Nicholson GM Hodgson WC (2008) Intersexual variations in Northern (*Missulena pruinosa*) and Eastern (*M. bradleyi*) mouse spider venom. Toxicon 51: 1167-1177. doi: 10.1016/j.toxicon.2008.02.001 18346773 10.1016/j.toxicon.2008.02.001

[B12] Isbister GK (2004) Mouse spider bites (*Missulena* spp.) and their medical importance. Medical Journal of Australia 180: 225-227 14984342

[B13] Main BY (1985) Mygalomorphae. In: Walton DW (Ed) Zoological Catalogue of Australia 3 Arachnida, Mygalomorphae, Araneomorphae in Part, Pseudoscorpionida, Amblypygida, Palpigradi. Australian Government Publishing Service, Canberra, 1-48

[B14] Main BY (1996) Biosystematics of Australian mygalomorph spiders: description of a new species of *Missulena* from southwestern Australia (Araneae: Mygalomorphae: Actinopodidae). Records of the Western Australian Museum Supplement 17: 355-359

[B15] Main BY Sampey A West PLJ (2000) Mygalomorph spiders of the southern Carnarvon Basin, Western Australia. Records of the Western Australian Museum, Supplement 61: 281-293

[B16] Mascord RE (1970) Australian spiders in colour. Reed, Sydney

[B17] McKenzie NL van Leeuwen S Pinder AM (2009) Introduction to the Pilbara Biodiversity Survey, 2002−2007. Records of the Western Australian Museum, Supplement 78: 3-89

[B18] Platnick NI (2014) The World Spider Catalog, Version 14.5. http://research.amnh.org/iz/spiders/catalog/INTRO1.html [accessed 8 April 2014]

[B19] Rash D Birinyi-Strachan LC Nicholson GM Hodgson WC (2000) Neurotoxic activity of venom from the Australian eastern mouse spider (*Missulena bradleyi*) involves modulation of sodium channel gating. British Journal of Pharmacology 130: 1817-1824. doi: 10.1038/sj.bjp.0703494 10952670 10.1038/sj.bjp.0703494 PMC1572261

[B20] Raven RJ (1985) The spider infraorder Mygalomorphae (Araneae): cladistics and systematics. Bulletin of the American Museum of Natural History 182: 1-180

[B21] Raven RJ Seeman O (2008) Spiders of the Greater Brisbane region. Queensland Museum, Brisbane, 68 pp.

[B22] Walckenaer CA (1805) Tableau des Aranéides ou caractères essentiels des tribus, genres, families et races que renferme le genre *Aranea* de Linné, avec la désignation des espèces comprises dans chacune de ces divisions. Dentu, Paris, 88 pp.

[B23] Walker KL Yen AL Milledge GA (2003) Spiders and scorpions commonly found in Victoria. Royal Society of Victoria, Melbourne, Vic, 144 pp.

[B24] Womersley H (1943) A revision of the spiders of the genus *Missulena* Walckenaer, 1805. Records of the South Australian Museum 7: 249-269

[B25] Wyrwoll K-H Stoneman T Elliott G Sandercock P (2000) The geoecological setting of the study area: geology, geomorphology and soils. Records of the Western Australian Museum, Supplement 61: 29-75

